# Operations for Breast Cancer

**Published:** 1898-02-19

**Authors:** 


### OPERATIONS FOR BREAST CANCER.

The discussion on this subject at the Royal Medical and Chirurgical Society continues to elicit from surgeons of wide experience confirmatory evidence of the fact that a very considerable number of cases of cancer are far more permanently cured of their disease than used to be thought possible some years ago. Another point also seems clear, namely, that it is not altogether by a rigid selection of cases that these successful results have been obtained. No doubt all modern knowledge tends to emphasise the advantages of early operation in cancer; nevertheless, the cases in which long intervals of immunity, or even cure, have resulted from operation have not been exclusivley those in which early operation has been done. Some of them, indeed, have been what we might call neglected cases. Mr. Jonathan Hutchinson said, in regard to this point, that his most successful cases had not always been those which appeared most promising, instancing one of a soft, rapidly-growing carcinoma in a young patient, which was freely removed fourteen years ago, the patient being quite well now, and another in which there was an ulcerated cavity into which the fist could have been placed, yet now, six years after removal, there was no recurrence. Mr. Barker also spoke to the same effect. In regard to the question of the extent of the operation which should be undertaken, it was shown that, while in some cases in which operation was manifestly "incomplete," or "inadequate," according to modern nomenclature, good results were obtained, it was impossible to be sure, even by the most complete operation, that all the infected material had been removed. Sir Thomas Smith pointed out that in the long interval which occurred between the first existence of cancer as a microscopic object in the breast and the period when it became a tangible tumour there was abundant time for constitutional infection to occur. He also made the very important suggestion that, necessary as it was to remove affected glands, it was possible that the removal of healthy glands might tend to promote recurrence.

In regard to the necessity for removing the pectoral muscles Mr. Howard Marsh differed somewhat from those who advocated the very large operations which were sometimes done now. The old partial operation was, he said, finally abandoned, but he thought the pendulum had swung a little into the opposite extreme. The point at which the operation was most likely to fail lay in the impossibility of completely removing the lymphatics in the immediate neighbourhood of the large vessels on a level with the clavicle. It was there, and not in the pectoral muscles, that recurrence was likely to take place, and it could not be denied that the extensive operation recommended by Halsted was attended with serious drawbacks, among which he mentioned shock—of which some patients had died—and, as a latter effect, fixation of the arm to the side with œdema of the hand and neuralgic pain. Still, if the operation should prove to secure a very long immunity these drawbacks must be accepted, but he did not think that at present this was the case.

